# Gut microbiota influence acute pancreatitis through inflammatory proteins: a Mendelian randomization analysis

**DOI:** 10.3389/fcimb.2024.1380998

**Published:** 2024-05-31

**Authors:** Peiyao Huang, Qiang Liu, Tianlong Zhang, Jianfeng Yang

**Affiliations:** ^1^ Department of Gastroenterology, the Fourth Affiliated Hospital of School of Medicine, and International School of Medicine, International Institutes of Medicine, Zhejiang University, Yiwu, China; ^2^ Department of Gastroenterology, Hangzhou First People’s Hospital, Zhejiang University School of Medicine, Hangzhou, China; ^3^ Key Laboratory of Integrated Traditional Chinese and Western Medicine for Biliary and Pancreatic Diseases of Zhejiang Province, Hangzhou, China; ^4^ Key Laboratory of Clinical Cancer Pharmacology and Toxicology Research of Zhejiang Province, Hangzhou, China; ^5^ Department of Critical Care Medicine, The Fourth Affiliated Hospital of School of Medicine, and International School of Medicine, International Institutes of Medicine, Zhejiang University, Yiwu, China

**Keywords:** acute pancreatitis, gut microbiota, inflammatory proteins, Mendelian randomization, single nucleotide polymorphism

## Abstract

**Background/Aim:**

We employed Mendelian randomization (MR) analysis to investigate the causal relationship between the gut microbiota, acute pancreatitis, and potential inflammatory proteins.

**Methods:**

The data for gut microbiota, acute pancreatitis, and inflammatory proteins are sourced from public databases. We conducted a bidirectional MR analysis to explore the causal relationship between gut microbiota and acute pancreatitis, and employed a two-step MR analysis to identify potential mediating inflammatory proteins. IVW is the primary analysis method, heterogeneity, pleiotropy, and sensitivity analyses were also conducted simultaneously.

**Results:**

We identified five bacterial genera associated with the risk of acute pancreatitis, namely *genus.Coprococcus3*, *genus.Eubacterium fissicatena group*, *genus.Erysipelotrichaceae UCG-003, genus.Fusicatenibacter, and genus.Ruminiclostridium6*. Additionally, we have discovered three inflammatory proteins that are also associated with the occurrence of acute pancreatitis, namely interleukin-15 receptor subunit alpha (IL-15RA), monocyte chemoattractant protein-4 (CCL13), and tumor necrosis factor receptor superfamily member 9 (TNFRSF9). Following a two-step MR analysis, we ultimately identified IL-15RA as a potential intermediate factor, with a mediated effect of 0.018 (95% CI: 0.005 - 0.032).

**Conclusion:**

Our results support the idea that *genus.Coprococcus3* promotes the occurrence of acute pancreatitis through IL-15RA. Furthermore, there is a potential causal relationship between the gut microbiota, inflammatory proteins, and acute pancreatitis. These findings provide new insights for subsequent acute pancreatitis prevention.

## 1 Introduction

Pancreatitis is a prevalent gastrointestinal disorder characterized by acute and chronic forms. A recent meta-analysis documented a worldwide prevalence of 33 cases per 100,000 person-years for acute pancreatitis (Xiao et al., 2016). Acute pancreatitis entails an inflammatory response, leading to self-digestion, edema, hemorrhage, and potentially necrosis of pancreatic tissues, triggered by the activation of pancreatic enzymes due to diverse etiological factors. Acute pancreatitis manifests as intense abdominal pain, nausea, vomiting, and various clinical symptoms, and in severe cases, it can lead to organ failure. Thus, it remains closely linked to high mortality, with two peaks of mortality, early and late (Garg and Singh, 2019).

There are 100 trillion microorganisms and more than 1000 different bacteria in the human intestine, which constitute the gut microbiota (Ramakrishna, 2013). Recent discoveries have shown that the gut microbiota is involved in regulating multiple host functions by producing bioactive bacterial metabolites. Therefore, it is considered a new human organ and also emerged as a key factor in the balance between health and disease (Baquero and Nombela, 2012; Lee and Hase, 2014; Schepis et al., 2021). It is now understood that dysregulation of the microbiota can lead to a range of diseases, including obesity, inflammatory bowel disease, Alzheimer’s disease, metabolic syndrome, cardiovascular disease, and even cancer (Sekirov et al., 2010; Distrutti et al., 2016; Brandi et al., 2021; Zhao et al., 2021).

The coordination of inflammatory responses involves a complex network of cells and mediators (Zhao et al., 2023), and dysregulated inflammatory proteins play a crucial role in disease progression. For example, elevated concentrations of interleukin-1α (IL-1α), interleukin-8 (IL-8), and tumor necrosis factor-α (TNF-α) are associated with an increased risk of ovarian cancer (Trabert et al., 2014). Since proteins serve as both effector molecules and drug targets in most biological processes, understanding their roles in diseases is a growing area of research.

Mendelian randomization (MR) analysis leverages genetic variation as instrumental variables (IVs) in place of exposure factors to evaluate the casual relationship between exposures and outcomes, mimicking random assignment in a study design (Wehby et al., 2008; Lee and Lim, 2019). This approach has gained popularity in recent epidemiological studies to overcome limitations inherent to observational and, to some extent, randomized controlled studies. Herein, we sought to explore the causal relationship between the gut microbiota and the onset risk of acute pancreatitis, investigating the role inflammatory proteins play in this process. Therefore, we conducted a two-step MR analysis based on genome-wide association study (GWAS) summary data.

## 2 Methods

### 2.1 Data sources

Genetic summary statistics for acute pancreatitis were generated from a GWAS data from FinnGen (GWAS ID: finn-b-K11_ACUTPANC), and for the human gut microbiome from the published meta-analysis by the MiBioGen consortium (Kurilshikov et al., 2021). We then excluded 15 bacterial traits lacking specific nomenclature, resulting in a final set of 196 bacterial traits, encompassing 9 phyla, 16 classes, 20 orders, 32 families, and 119 genera. The data on inflammatory proteins were obtained from Jing et al (Zhao et al., 2023), who conducted a genome-wide protein quantitative trait locus (pQTL) study of 91 inflammatory proteins measured using the Olink Target platform in 14,824 European-ancestry participants. As the present study was based on public summary data, no additional ethics approval or consent to participate was required.

### 2.2 Instrumental variable selection

IVs were chosen at a significance level of p< 1.0 × 10^-5^. To ensure independence of the IVs from loci, we applied a linkage disequilibrium (LD) threshold of R^2^< 0.001 and a clumping distance of 10,000 kb in the 1000 Genomes European (EUR) data using the “TwoSampleMR” packages. Additionally, we extracted relevant information for each single nucleotide polymorphism (SNP), including effect alleles, β-value, standard deviation, and p-values. We then calculated the proportion of variation explained (R^2^) and F value to quantify instrument strength using the following equation:


$${\text{R}}^{2}=\frac{2\times {\text{β}}^{2}\times \text{EAF}\times (1-\text{EAF})}{2\times {\text{β}}^{2}\times \text{EAF}\times \left(1-\text{EAF}\right)+2\times \text{S}{\text{E}}^{2}\times \text{N}\times \text{EAF}\times \left(1-\text{EAF}\right)}$$

$$\text{F}=\frac{{\text{R}}^{2}\times (\text{N}-2)}{\left(1-{\text{R}}^{2}\right)}$$

Where “N” is the number of participants, “EAF” is the effect allele frequency, “β” is the estimated effect of the SNP (assessing its ability to uniquely predict the outcome), and “SE” is the standard error of the genetic effect (Palmer et al., 2012; Gill et al., 2019; Levin et al., 2020). A higher F value (greater than 10) indicates a lower likelihood of weak instrument bias.

### 2.3 Statistical analysis

**Figure 1:**
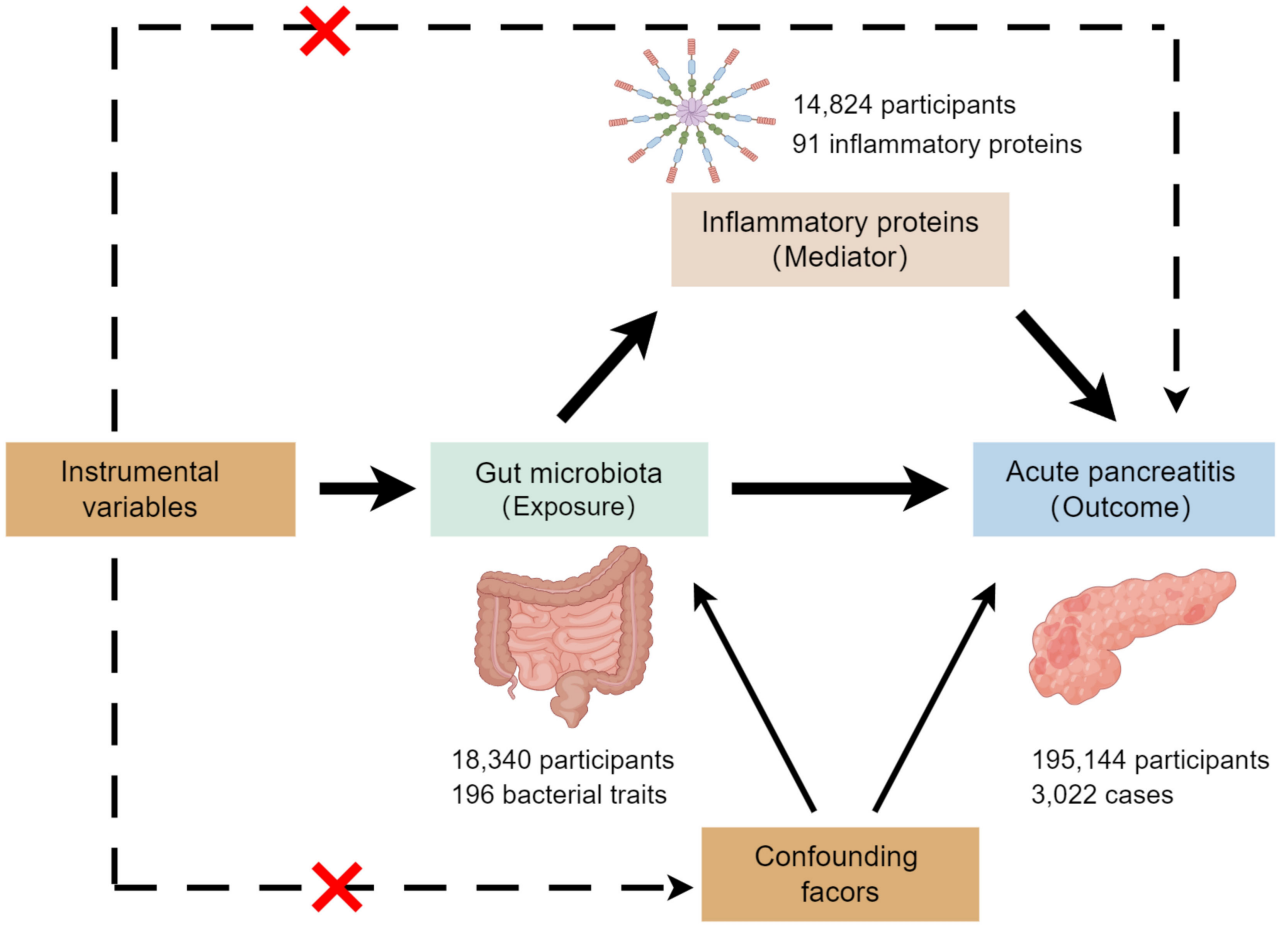
The study design. By Figdraw.

This study employed a two-sample MR analysis to investigate the association between exposures (gut microbiota) and outcomes (the risk of acute pancreatitis). Additionally, a two-step MR analysis was conducted to assess whether inflammatory proteins mediate the potential effect of gut microbiota on pancreatitis development. **Figure 1** illustrates the overall design of the study.

In this study, we employed various MR analysis methods, including inverse variance weighting (IVW), MR-Egger regression, weighted median, weighted model, and simple model. Among these, IVW provides the most precise overall effect estimate by combining a meta-analysis approach with the estimates of the effect of each genetic variant for gut microbiota on pancreatitis (Burgess et al., 2016; Li et al., 2022). Therefore, we used IVW as the primary analysis method. We conducted a series of MR analyses: first, a two-sample MR analysis for gut microbiota and acute pancreatitis. Second, a reverse MR analysis on bacteria causally associated with pancreatitis from the first analysis. Third, an MR analysis of these bacteria and 91 inflammatory proteins. Finally, we explored the relationship between inflammatory proteins and acute pancreatitis. Throughout the analyses, we employed Cochran’s Q test to assess the heterogeneity of IVs, MR-PRESSO and MR-Egger regression to check for potential horizontal pleiotropy, and the “leave-one-out” method for sensitivity analysis. P values < 0.05 were considered nominally significant. All MR analyses were performed using R version 4.2.2 (https://www.r-project.org/). MR analyses were performed using the “TwoSampleMR” package and MRPRESSO package.

## 3 Results

### 3.1 Total effect of gut microbiota on acute pancreatitis

**Figure 2:**
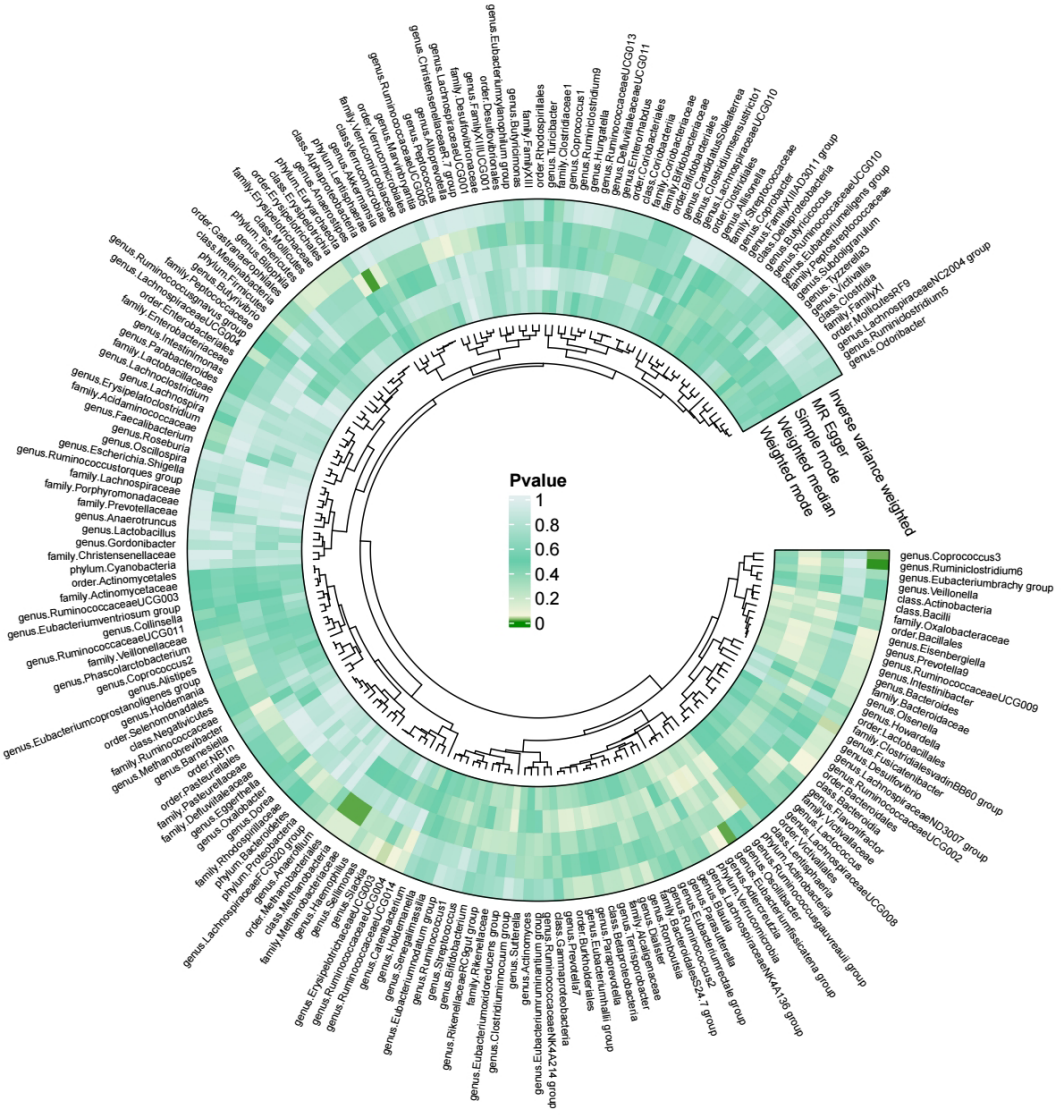
Circos plot of the relationship between gut microbiota and acute pancreatitis.

**Figure 3:**
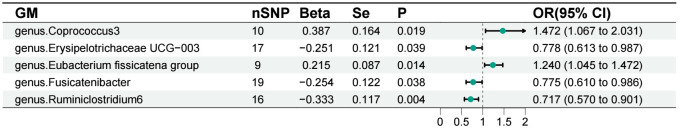
Forest plot of the associations between gut microbiota and acute pancreatitis. GM, gut microbiota.

Our results were visualized in a Circos plot (**Figure 2**). Five bacterial genera showed potential associations with acute pancreatitis. Using the IVW method, genetic predisposition to *genus.Coprococcus3* and *genus.Eubacterium fissicatena group* may be associated with an increased risk of acute pancreatitis. Conversely, *genus.Erysipelotrichaceae UCG-003*, *genus.Fusicatenibacter*, and *genus. Ruminiclostridium6* were protective against acute pancreatitis (**Figure 3**). Heterogeneity and pleiotropy tests yielded p-values > 0.05, indicating consistency in the results. Sensitivity analysis yielded robust and consistent results (**Supplementary 1**, **2**).

### 3.2 Reverse MR analysis

Next, we evaluated the potential reverse associations of bacterial traits and acute pancreatitis using the reverse MR analyses. With the IVW method, no significant causal association was found between acute pancreatitis and any of these bacterial traits (**Table 1**). The results remained stable across sensitivity analyses.

**Table 1:** Reverse MR analysis of acute pancreatitis and gut microbiota.

GM	nSNP	OR	95% CI	P
genus.Coprococcus 3	7	1.009	0.933 to 1.091	0.820
genus.Erysipelotrichaceae UCG-003	1	0.839	0.699 to 1.008	0.061
genus.Eubacterium fissicatena group	7	1.072	0.861 to 1.335	0.531
genus.Fusicatenibacter	7	1.005	0.944 to 1.070	0.868
genus.Ruminiclostridium 6	7	0.983	0.910 to 1.064	0.683

GM, gut microbiota.

### 3.3 Casual effect of gut microbiota on inflammatory proteins

**Figure 4:**
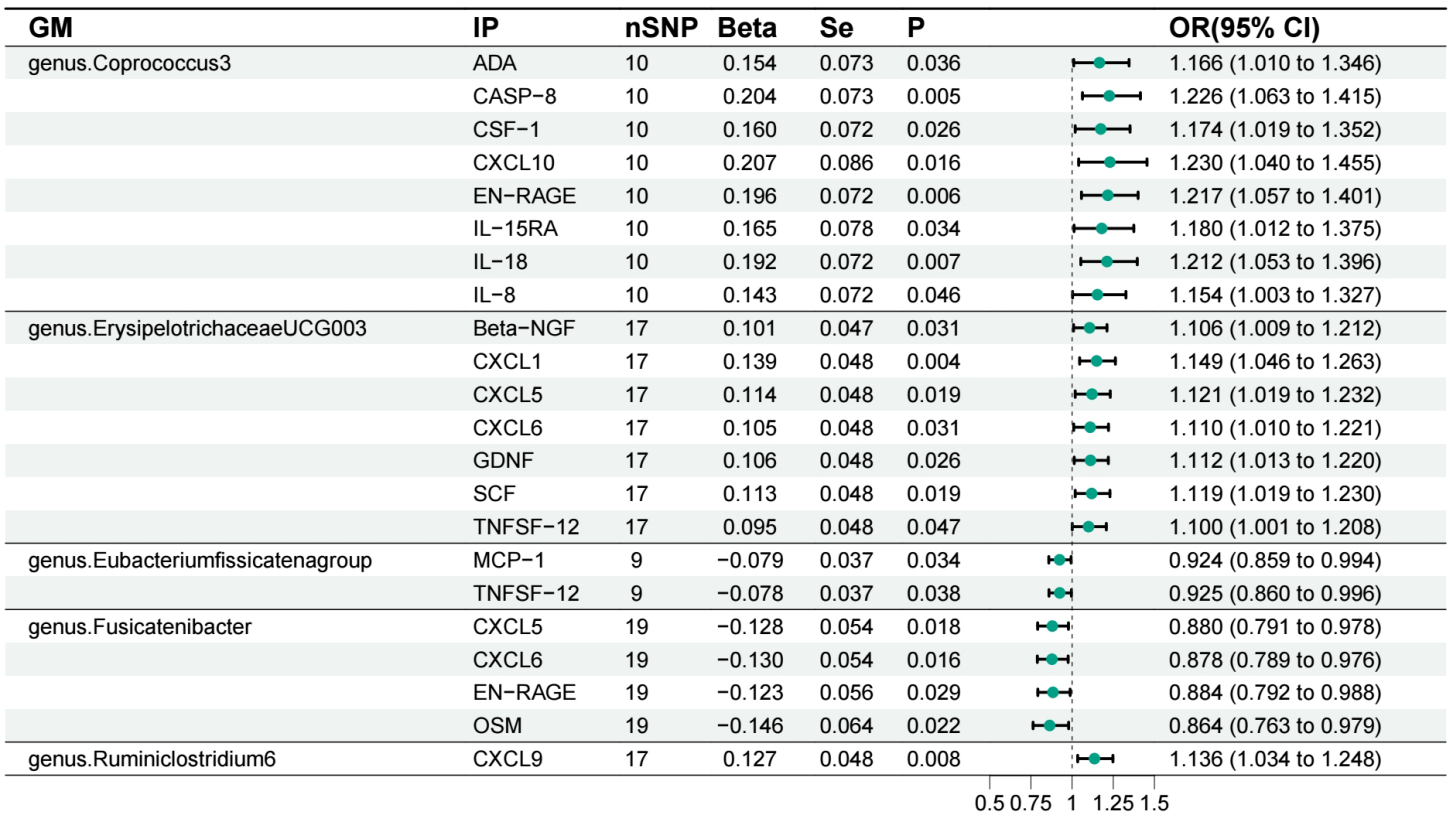
Forest plot of the associations between gut microbiota and inflammatory proteins. GM, gut microbiota; IP, inflammatory protein; ADA, adenosine deaminas; CASP-8, caspase-8; CSF-1, macrophage colony-stimulating factor-1; CXCL10, C-X-C motif chemokine-10; EN-RAGE, protein S100-A1; IL-15RA, Interleukin-15 receptor subunit alpha; IL-18, Interleukin-18; IL-8 Interleukin-8; Beta-NGF, beta-nerve growth factor; CXCL1, C-X-C motif chemokine-1; CXCL5, C-X-C motif chemokine-5; CXCL6, C-X-C motif chemokine-6; GDNF, Glial cell line-derived neurotrophic factor; SCF, Stem cell factor; TNFSF12, Tumor necrosis factor ligand superfamily member 12; MCP-1, monocyte chemoattractant protein-1; OSM, Oncostatin-M; CXCL9, C-X-C motif chemokine 9.

Our findings based on gene prediction suggest that a positive association between increased abundance of *genus.Coprococcu3* and several inflammatory proteins, including adenosine deaminase (ADA), caspase-8 (CASP-8), macrophage colony-stimulating factor-1 (CSF-1), C-X-C motif chemokine-10 (CXCL10), S100A1 protein (EN-RAGE), interleukin-15 receptor subunit alpha (IL-15RA), interleukin-18 (IL-18) and interleukin-8 (IL-8). Similarly, an increase in *genus.ErysipelotrichaceaeUCG003* abundance was associated with increased levels of beta-nerve growth factor (Beta-NGF), C-X-C motif chemokine-1 (CXCL1), C-X-C motif chemokine-5 (CXCL5), C-X-C motif chemokine-6 (CXCL6), glial cell line-derived neurotrophic factor (GDNF), stem cell factor (SCF) and tumor necrosis factor ligand superfamily member 12 (TNFSF12). The *genus.Ruminiclostridium6*, on the other hand, showed a positive correlation with C-X-C motif chemokine 9 (CXCL9). Conversely, increased *genus.Eubacterium fissicatena group* abundance was associated with reduced levels of inflammatory proteins such as monocyte chemoattractant protein-1 (MCP-1) and TNFSF-12. *Genus.Fusicatenibacter* also had a negative impact on CXCL5, CXCL6, EN-RAGE and oncostatin-M (OSM) (**Figure 4**). Importantly, no significant heterogeneity or pleiotropy was observed in our data (**Supplementary 1**, **2**).

### 3.4 Causal effect of inflammatory proteins on acute pancreatitis

**Figure 5:**
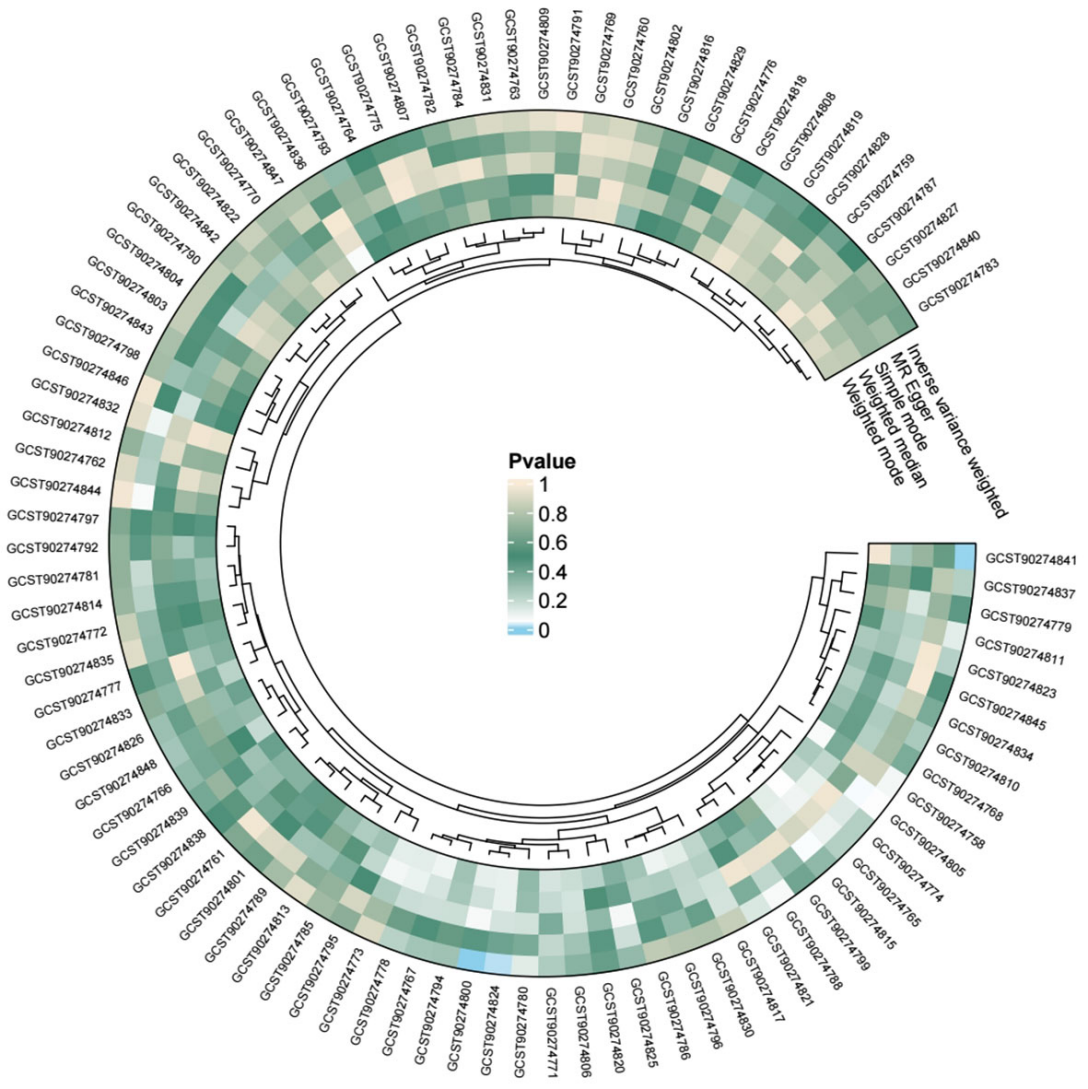
Circos plot of the relationship between inflammatory proteins and acute pancreatitis.

**Figure 6:**
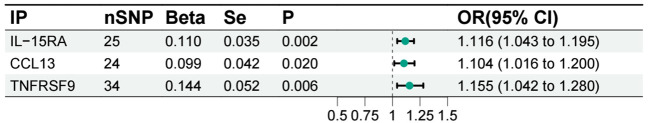
Forest plot of the associations between inflammatory proteins and acute pancreatitis. IP, inflammatory protein; IL-15RA, Interleukin-15 receptor subunit alpha; CCL13, Monocyte chemoattractant protein-4; TNFRSF9, Tumor necrosis factor receptor superfamily member 9.

We conducted individual analyses of 91 inflammatory proteins concerning acute pancreatitis, and all the results are presented in the following figure (**Figure 5**). Among these, genetically predicted IL-15RA, monocyte chemoattractant protein-4 (CCL13), and tumor necrosis factor receptor superfamily member 9 (TNFRSF9) showed a significant positive correlation with the occurrence of acute pancreatitis (**Figure 6**).

### 3.5 Mediation effect of IL-15RA

**Figure 7:**
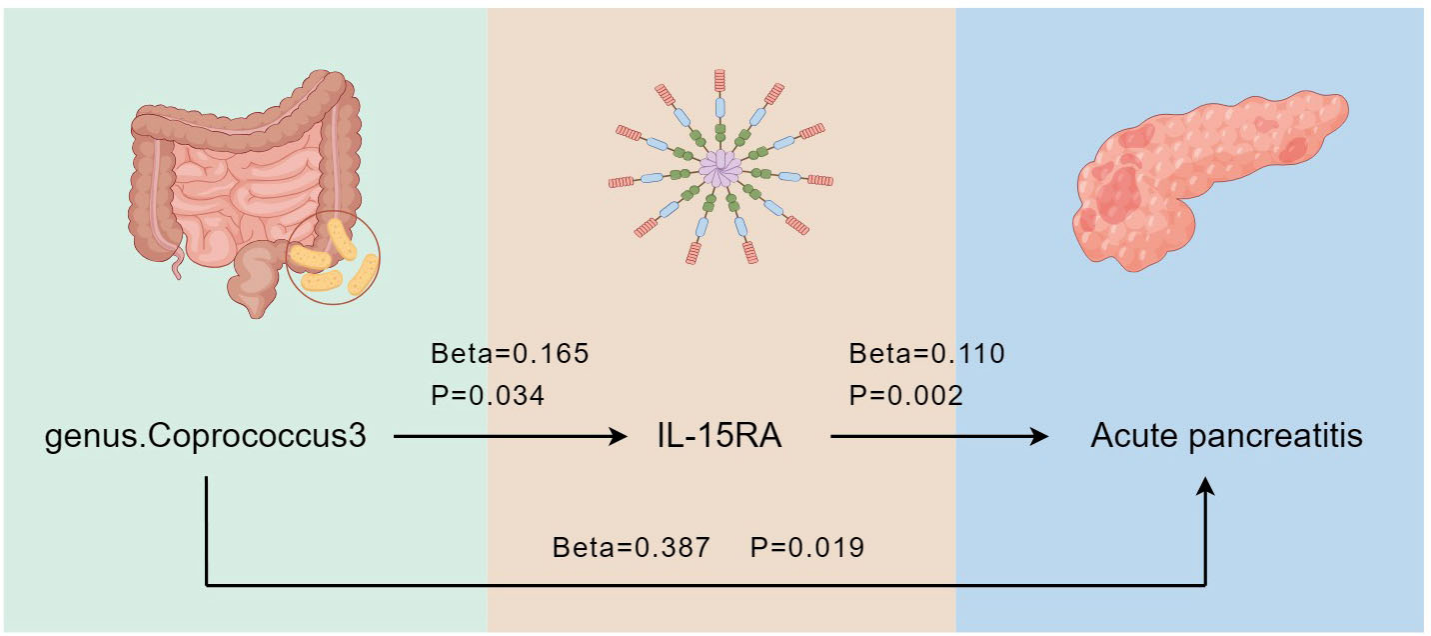
Effects of IL-15RA on gut microbiota and acute pancreatitis. By Figdraw.

Our two-step MR analysis, identified IL-15RA as a crucial intermediate factor influencing the relationship between *genus.Coprococcus3* and acute pancreatitis (**Figure 7**). We found that *genus.Coprococcus3* could increase the risk of acute pancreatitis by positively influencing IL-15RA. The overall effect of *genus.Coprococcus3* on acute pancreatitis was 0.387, with a direct effect of 0.369. IL-15RA has a mediating effect of 0.018 (95%CI: 0.005 - 0.032), indicating that 4.713% of the effect of *genus.Coprococcus3* on pancreatitis was mediated by IL-15RA.

## 4 Discussion

Our present study investigated the causal link between gut microbiota, inflammatory proteins, and the risk of acute pancreatitis using genetic prediction. We revealed that IL-15RA plays a mediating role in how *genus.Coprococcus3* influences the development of acute pancreatitis. Furthermore, we identified associations between distinct microbial communities and specific inflammatory proteins associated with the disease.

IL-15RA is a high-affinity binding protein for interleukin-15 (IL-15). Its structure includes a signal peptide, sushi domain, hinge region, proline-threonine rich region, transmembrane domain and cytoplasmic domain (Schluns et al., 2005). Expressed in various cell types, IL-15RA plays a crucial role in mediating IL-15 function and T-cell biology (Giri et al., 1995). Studies have shown that the main signaling mechanism for memory T cell survival and proliferation *in vivo* involves the trans-delivery of IL-15 by IL-15RA on hematopoietic and non-hematopoietic cell types (Burkett et al., 2003; Burkett et al., 2004; Kenesei et al., 2021). Rowley et al. (2009) found that IL-15RA can transparent IL-15 in a cis manner to T cells, promoting the phosphorylation of signal transducer and activator of transcription-5 (STAT5), and the survival and proliferation of unstimulated CD8^+^ T cells. Moreover, IL-15RA expression alone promotes cell-autonomous survival and proliferation of primary unstimulated CD8^+^ T cells both *in vitro* and *in vivo*. Additionally, it increases proliferation and interferon-γ (IFN-γ) production in antigen-specific T cells *in vitro*. No prior research has investigated the relationship between IL-15RA and acute pancreatitis or *genus.Coprococcus*. However, elevated levels of IL-15RA play a crucial role in the pathogenesis of systemic lupus erythematosus (SLE), rheumatoid arthritis (RA), ulcerative colitis (UC), and Crohn’s disease (CD) (Baranda et al., 2005; MaChado Diaz et al., 2012; Jadidi et al., 2022). Patients with UC and CD exhibit heightened mucosal expression of IL-15RA, accompanied by increased serum levels in UC individuals (Perrier et al., 2013). Interestingly, IL-15RA is also thought to harbor anti-tumor effects. The IL-15RA rs2228059 A > C polymorphism decreased the risk of gastric cardiac adenocarcinoma and esophageal cancer in a Chinese population (Yin et al., 2014a; Yin et al., 2014b). Aerobic exercise can further promote immune mobilization and the accumulation of tumor-infiltrating IL15RA and CD8^+^ T cells, thereby exerting anti-tumor effects (Kurz et al., 2022). Existing research has shown that serum levels of IL-15 significantly increase in patients with severe acute pancreatitis, and it can be used for predicting complications and mortality associated with severe acute pancreatitis (Ueda et al., 2007). While the inflammatory protein data analyzed in our study lacks information on IL-15 itself, IL-15RA serves as the specific high-affinity receptor for IL-15. Upon binding, they activate JAK-STAT5 signal transduction molecules, subsequently leading to the activation of multiple signaling pathways. We speculate that these interactions between IL-15 and IL-15RA may have an impact on pancreatic tissue, although the specific mechanisms require further investigation.

Several studies have explored the relationship between specific gut bacteria and various health conditions. For instance, Alferink et al. conducted a large-scale cross-sectional study that found a weak correlation between *Coprococcus* and steatosis (Alferink et al., 2021). MR studies have shown that *Coprococcus3* is associated with an increased risk of obstructive sleep apnea and cholelithiasis (Liu et al., 2023; Wei et al., 2023). Palm et al. observed that a member of the *Erysipelotrichaceae* family exhibits higher immunoglobulin A (IgA) coating compared to other gut microbes (Palm et al., 2014). Additionally, the relative abundance of *Erysipelotrichi* positively correlates with TNF-α (Dinh et al., 2015). In addition, *Erysipelotrichaceae* may also be related to metabolism. Earlier studies showed increased levels of *Erysipelotrichaceae* in diet-induced obese mice (Turnbaugh et al., 2008; Fleissner et al., 2010). Higher levels of *Erysipelotrichaceae* have also been observed in obese individuals, as well as smoking population (Zhang et al., 2009; Kaakoush, 2015; Yang et al., 2022). Several recent MR studies have yielded interesting, and sometimes seemingly contradictory, findings on specific gut bacteria. The *Eubacterium fissicatena group*, for example, has been shown to have a significant negative causal effect on appendicular lean mass (Zhao et al., 2023) while exhibiting a positive correlation with psoriasis (Zang et al., 2023). Animal experiments by Chonghui Yue et al. (2020) further supported this complex interplay, demonstrating a negative correlation between *Eubacterium fissicatena group* with various parameters, including epididymal white adipose tissue weight (eWAT weight), TNF-α, interleukin-6 (IL-6), MCP-1 in serum inflammation factors and recombinant peroxisome proliferator-activated receptor-γ (PPARγ), acetyl CoA carboxylase (ACC) in lipid metabolism-related proteins. Interestingly, some traditional Chinese medicines like Sophora japonica flowers, compound Chenpi tea, Gegen Qinlian decoction and Banxia Xiexin decoction have been linked to reducing the abundance of this bacterial group (Chen et al., 2021; Liu et al., 2022; Li et al., 2022; Wang et al., 2024). *Fusicatenibacter* on the other hand, plays a beneficial role. Known for producing short-chain fatty acids (Gryaznova et al., 2023) and degrading inulin polysaccharides, it also secretes interleukin-10 (IL-10), an anti-inflammatory cytokine (Takada et al., 2013; Takeshita et al., 2016). IL-10, a negative-feedback regulator cytokine, inhibits production of inflammatory cytokines like interleukin-1 (IL-1), TNF-α, and interleukin-12 (IL-12) from macrophages and suppresses T cell activation by inhibiting the expression of costimulators and MHCII on macrophages and dendritic cells (DCs) (Sziksz et al., 2015). Studies in Chinese population suggest a correlation between IL-10 and acute pancreatitis occurrence (Jia et al., 2015; Li et al., 2015; Jiang et al., 2016). Additionally, pirfenidone has been shown to augment the IL-10-driven anti-inflammatory pathway in macrophages, contributing to its effectiveness in treating acute pancreatitis (Palathingal Bava et al., 2022). While *Ruminiclostridium6* is not well-studied, some MR analyses associate it with the risk of moderate to severe asthma, scoliosis, and breast inflammatory disease (Gu et al., 2023; Lai et al., 2023; Li et al., 2023). Over the past few decades, research has increasingly revealed the “gut microbiota-pancreatic axis”, highlighting the mutual influence between gut bacteria and the pancreas (Schepis et al., 2021). Under pathological conditions, bacterial translocation to the pancreas can occur. As the intestinal microbial load increases and the epithelial barrier weakens, pancreatitis severity worsens (Zheng et al., 2019; Zhang et al., 2022). Studies comparing with healthy individuals to patients with pancreatitis have shown a decrease in bacterial phyla diversity in the latter group. Specifically, *Bacteroidetes*, *Proteobacteria*, *Enterococcus*, and *Enterobacteriaceae* were found to be more abundant, while *Firmicutes*, *Actinobacteria*, *Prevotella9*, *Baculobacter*, *Brucella*, *Lactospiraceae*, and *Bifidobacterium* were less common (Zhang Xi et al., 2018; Zhu et al., 2018). However, our MR analysis did not identify a causal relationship between these specific bacteria and acute pancreatitis. Indeed, it should be borne in mind that MR is a genetic approach that explores potential relationships between exposures and outcomes, and differs from observational studies in its methodology.

Our MR analysis has identified a causal relationship between gut microbiota and pancreatitis, revealing the mediating role of inflammatory proteins. This approach offers new insights for future research by mitigating the influence of confounding factors and revealing a novel genetic link between these factors. From a disease prevention perspective, these findings suggest the potential for preventing pancreatitis by gut microbiota, inflammatory proteins, or relevant factors through timely adjustments. However, the limitations of the present study should be acknowledged. The majority of individuals participating in the GWAS gut microbiota coefficient meta-analysis were of European ancestry, potentially limiting the generalizability of our results to non-European populations. Furthermore, the absence of data stratified by different etiologies of pancreatitis prevented consideration of etiological influences, thereby restricting our comprehensive investigation into how various causes may impact the observed genetic associations. Additionally, future research is necessary to elucidate the exact mechanisms, targets, and pathways underlying this association. Therefore, caution is warranted when interpreting the current findings.

## 5 Conclusion

MR analysis revealed that *genus. Coprococcus3* promotes acute pancreatitis through IL-15RA. Other intestinal bacteria, such as the *genus.Eubacterium fissicatena group*, are associated with an increased risk of acute pancreatitis, while, *genus.Ruminiclostridium6*, *genus.Fusicatenibacter*, and *genus.Erysipelotrichaceae UCG-003* appear to be protective factors. Inflammatory proteins CCL13 and TNFRSF9 also play a promoting role in the development of acute pancreatitis. These findings may provide insights for preventing acute pancreatitis, but the specific mechanisms require further investigation.

## Data availability statement

The original contributions presented in the study are included in the article/**Supplementary Materials**, further inquiries can be directed to the corresponding author/s.

## Author contributions

PH: Formal analysis, Visualization, Writing – original draft. QL: Data curation, Formal analysis, Writing – review & editing. TZ: Formal analysis, Visualization, Writing – original draft. JY: Conceptualization, Funding acquisition, Project administration, Writing – review & editing.
