# Supplementary material for: Gut microbiota influence acute pancreatitis through inflammatory proteins: a Mendelian randomization analysis
Source: Front Cell Infect Microbiol. 2024 May 31;14:1380998. doi: 10.3389/fcimb.2024.1380998 (PMC11176513; doi:10.3389/fcimb.2024.1380998)

genus.Coprococcus3 – acute pancreatitis

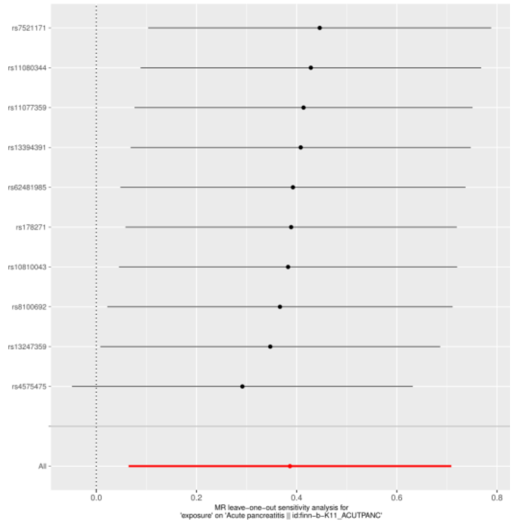

genus.Fusicatenibacter - acute pancreatitis

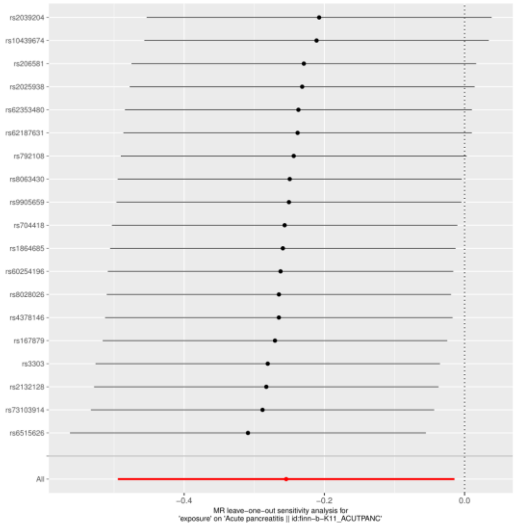

genus.ErysipelotrichaceaeUCG003 – acute pancreatitis

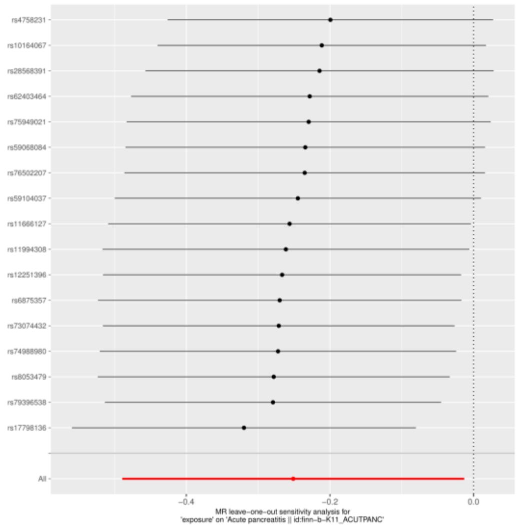

genus.Ruminiclostridium6 – acute pancreatitis

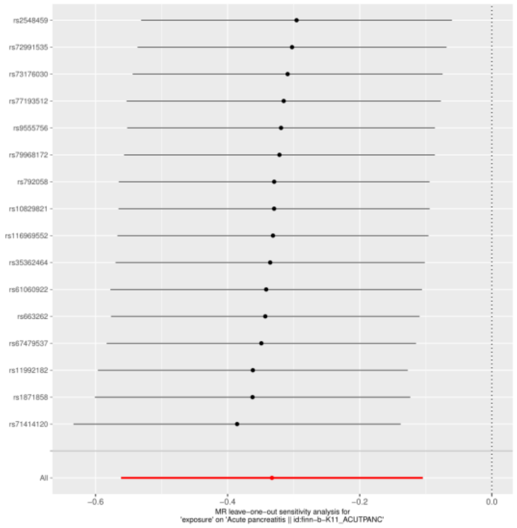

genus.Eubacteriumfissicatenagroup – acute pancreatitis

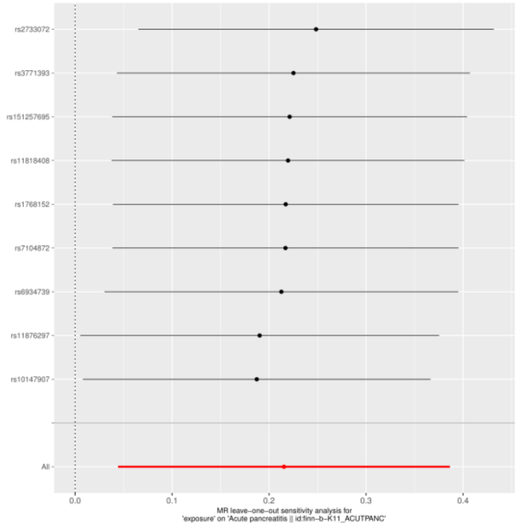

genus.Coprococcus3 - ADA

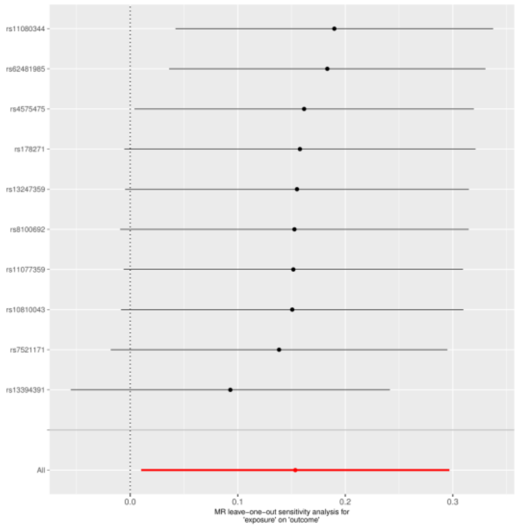

genus.Coprococcus3 – CXCL10

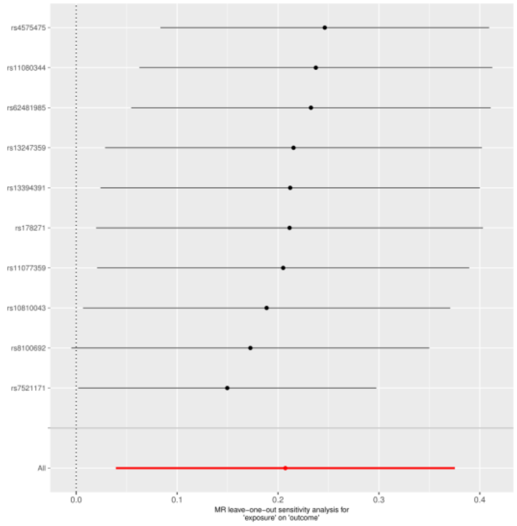

genus.Coprococcus3 – CASP-8

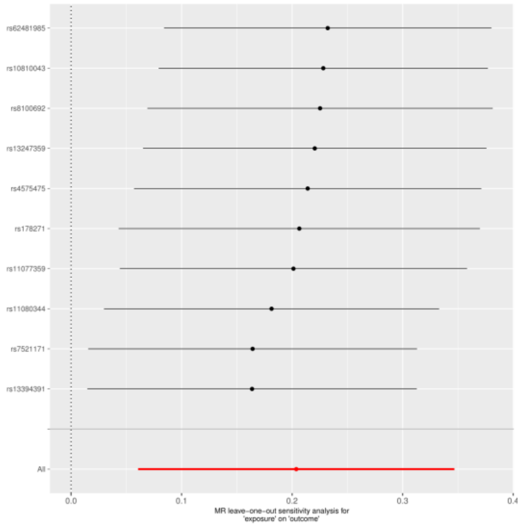

genus.Coprococcus3 – EN-RAGE

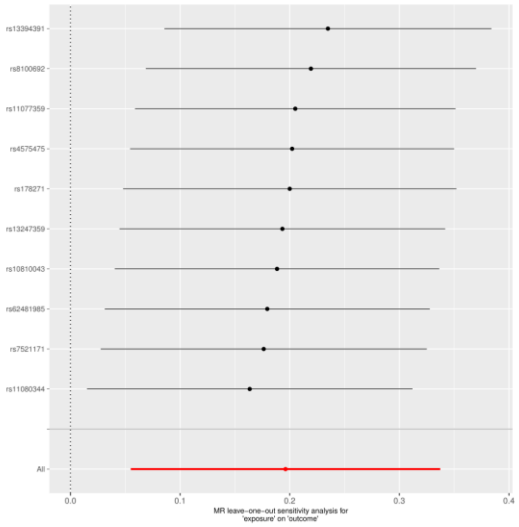

genus.Coprococcus3 – CSF-1

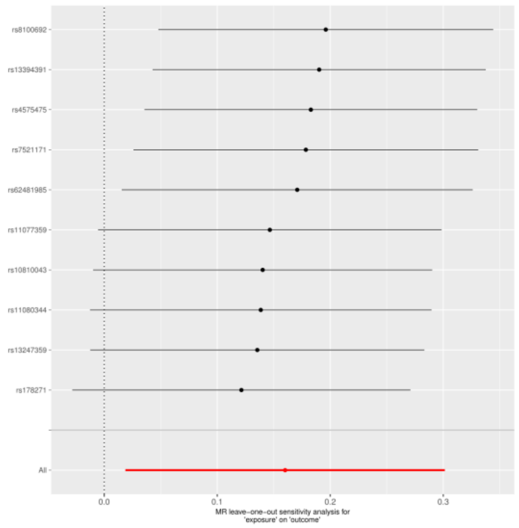

genus.Coprococcus3 – IL-15RA

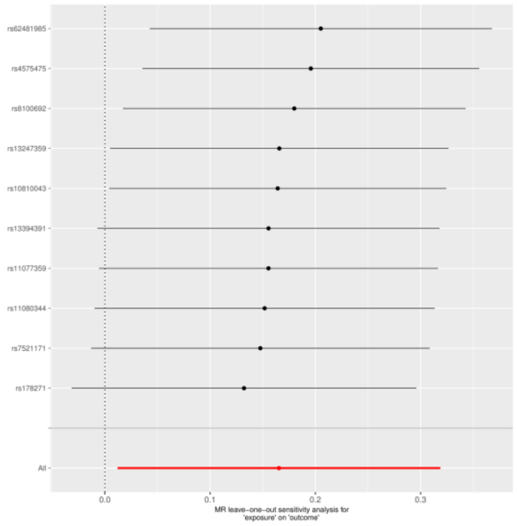

genus.Coprococcus3 – IL-18

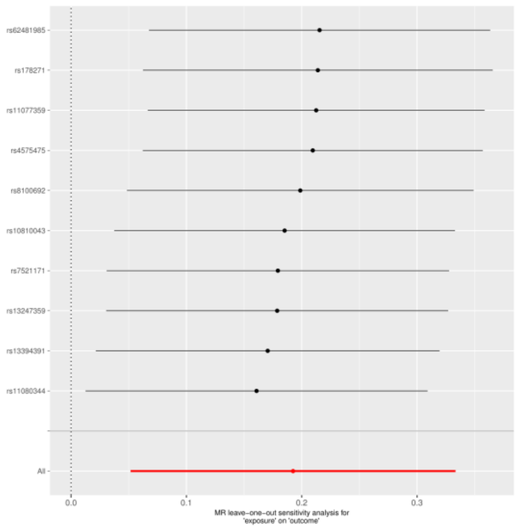

genus.ErysipelotrichaceaeUCG003 – CXCL1

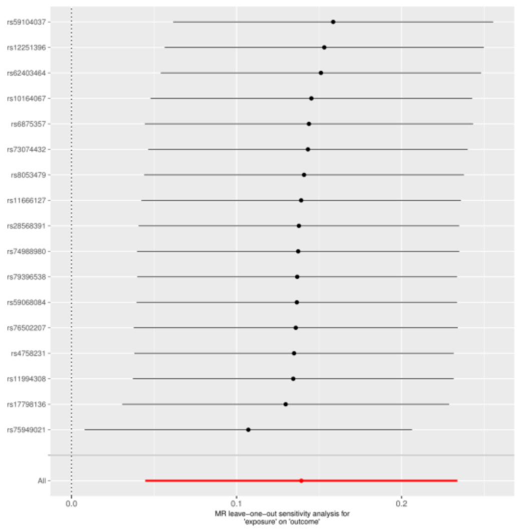

genus.Coprococcus3 – IL-8

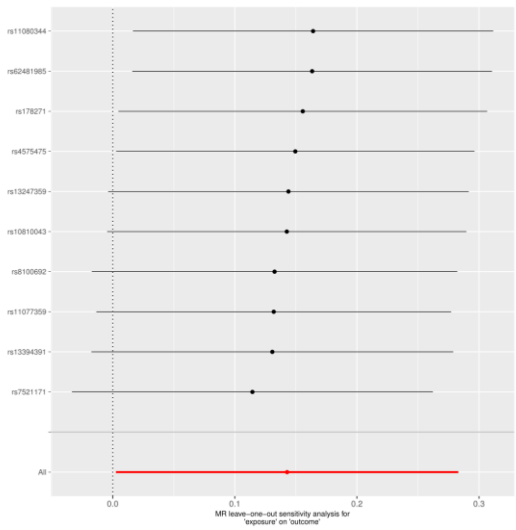

genus.ErysipelotrichaceaeUCG003 – CXCL5

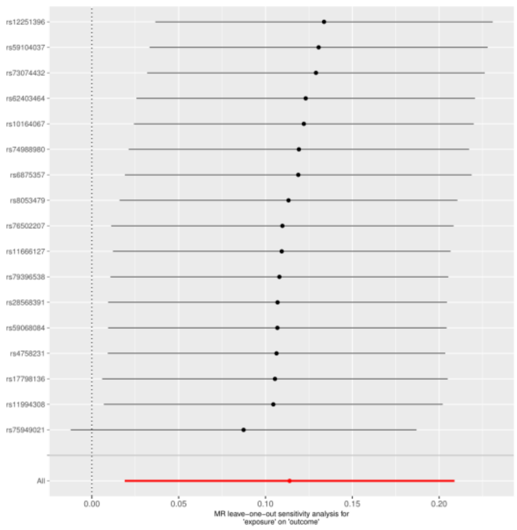

genus.ErysipelotrichaceaeUCG003 – Beta-NGF

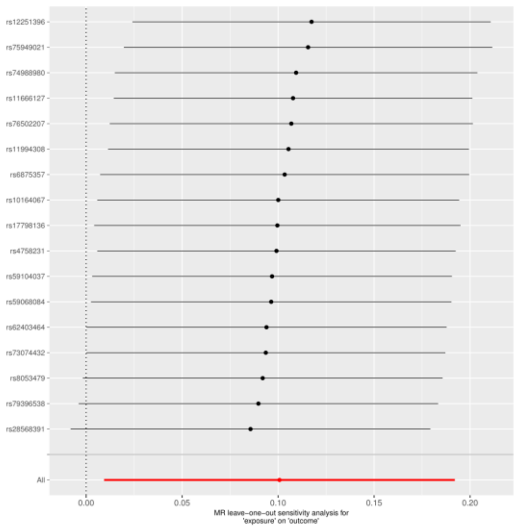

genus.ErysipelotrichaceaeUCG003 – CXCL6

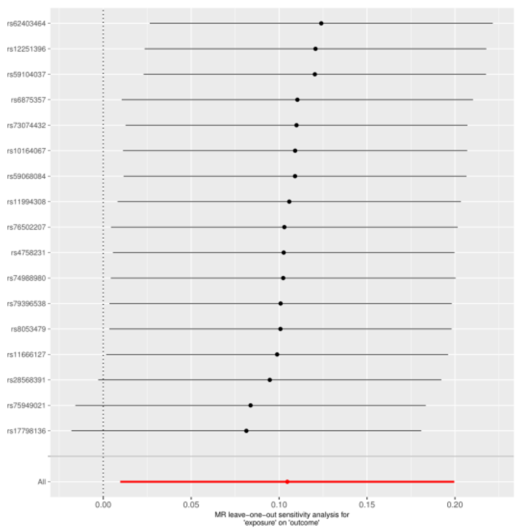

genus.ErysipelotrichaceaeUCG003 – GDNF

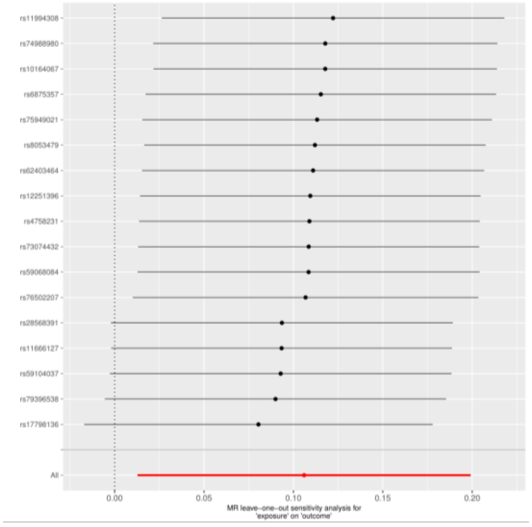

genus.Eubacteriumfissicatenagroup – MCP-1

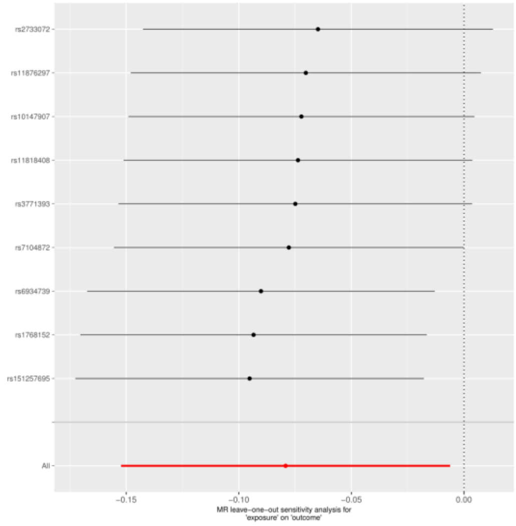

genus.ErysipelotrichaceaeUCG003 – SCF

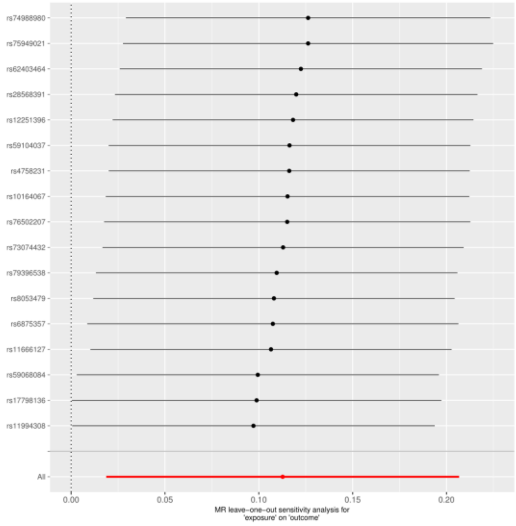

genus.Eubacteriumfissicatenagroup –TNFSF-12

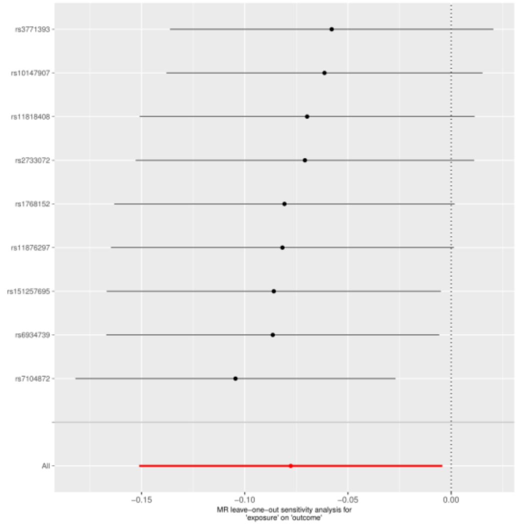

genus.ErysipelotrichaceaeUCG003 – TNFSF-12

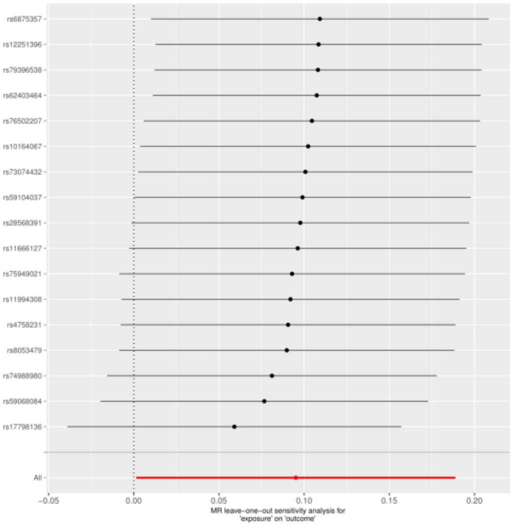

genus.Fusicatenibacter – CXCL5

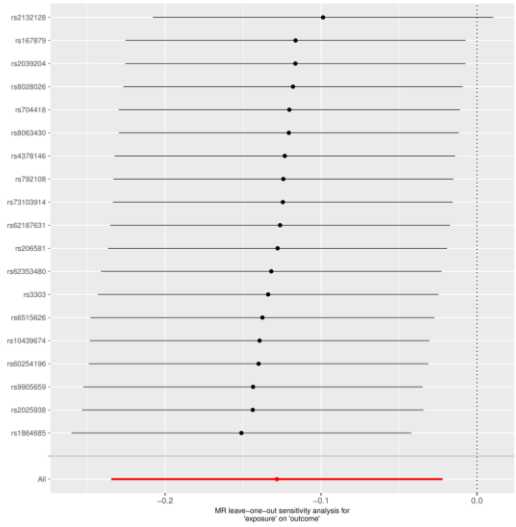

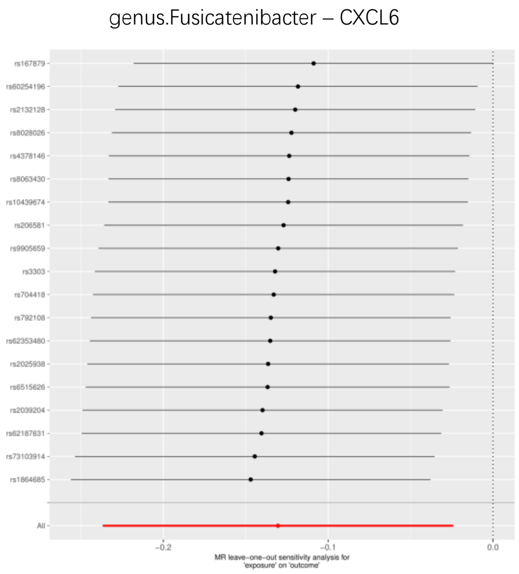
genus.Fusicatenibacter – CXCL6
rs167879
rs60254196
rs2132128
rs8028026
rs4378146
rs8063430
rs10439674
rs206581
rs9905659
rs3303
rs704418
rs792108
rs62353480
rs2025938
rs6515626
rs2039204
rs62187631
rs73103914
rs1864685
All
−0.2
−0.1
0.0
MR leave−one−out sensitivity analysis for
'exposure' on 'outcome'

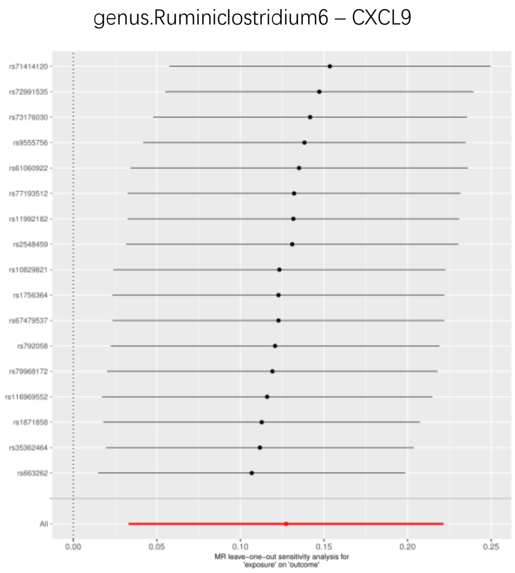
genus.Ruminiclostridium6 – CXCL9
rs71414120
rs72991535
rs73176030
rs9555756
rs61060922
rs77193512
rs11992182
rs2548459
rs10829821
rs1756364
rs67479537
rs792058
rs79968172
rs116969552
rs1871858
rs35362464
rs663262
All
0.00
0.05
0.10
0.15
0.20
0.25
MR leave−one−out sensitivity analysis for
'exposure' on 'outcome'

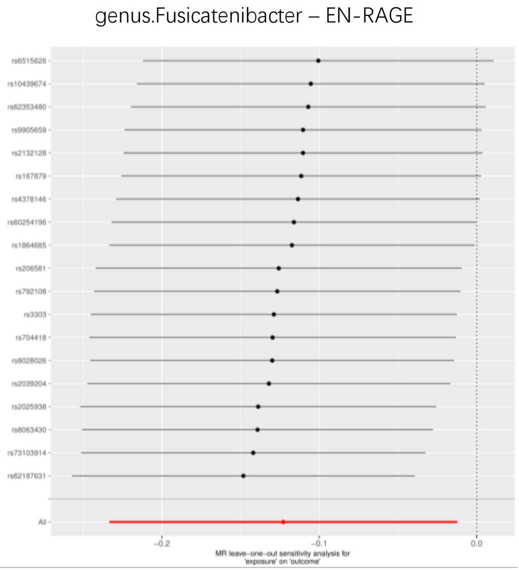
genus.Fusicatenibacter – EN-RAGE
rs6515626
rs10439674
rs62353480
rs9905659
rs2132128
rs167879
rs4378146
rs60254196
rs1864685
rs206581
rs792108
rs3303
rs704418
rs8028026
rs2039204
rs2025938
rs8063430
rs73103914
rs62187631
All
−0.2
−0.1
0.0
MR leave−one−out sensitivity analysis for
'exposure' on 'outcome'

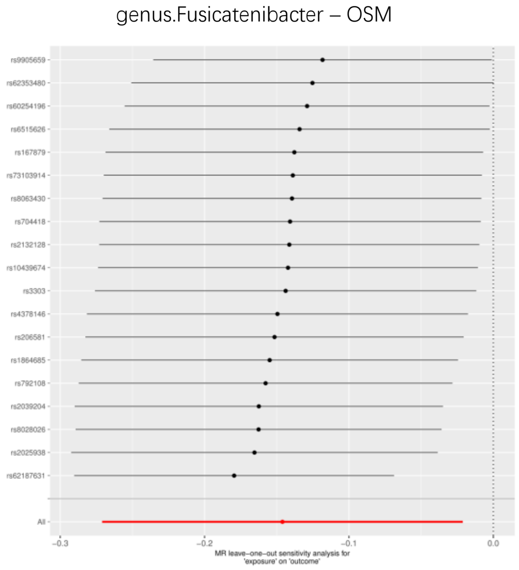
genus.Fusicatenibacter – OSM
rs9905659
rs62353480
rs60254196
rs6515626
rs167879
rs73103914
rs8063430
rs704418
rs2132128
rs10439674
rs3303
rs4378146
rs206581
rs1864685
rs792108
rs2039204
rs8028026
rs2025938
rs62187631
All
−0.3
−0.2
−0.1
0.0
MR leave−one−out sensitivity analysis for
'exposure' on 'outcome'

IL-15RA – acute pancreatitis

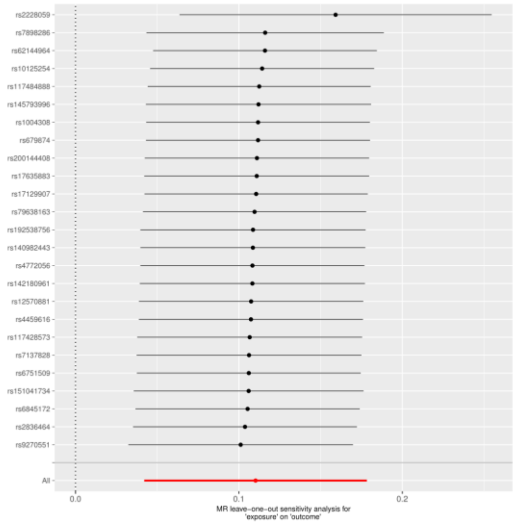

CCL13 – acute pancreatitis

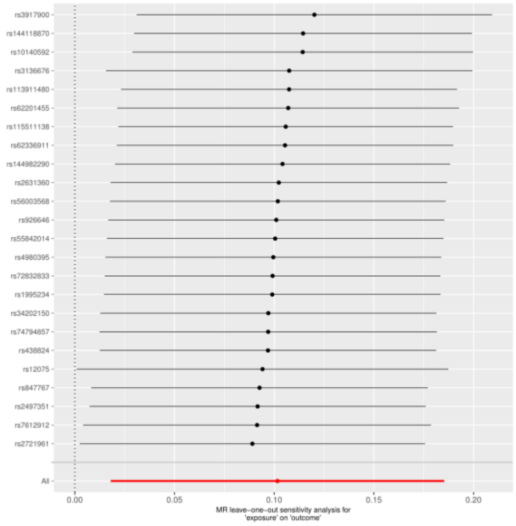

TNFRSF9 – acute pancreatitis

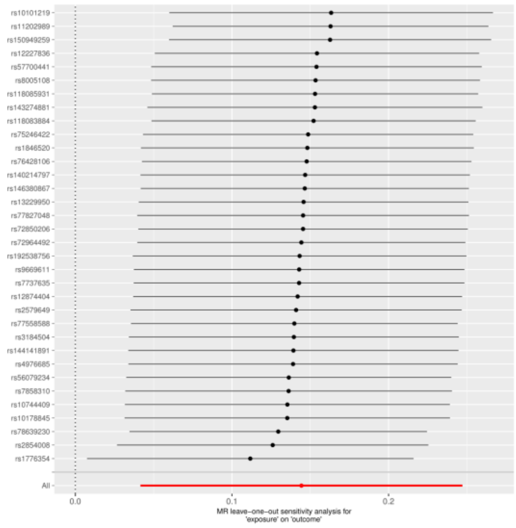

Supplement: Supplementary file 1 [file DataSheet_1.pdf]
